# Prokaryotic Solute/Sodium Symporters: Versatile Functions and Mechanisms of a Transporter Family †

**DOI:** 10.3390/ijms22041880

**Published:** 2021-02-13

**Authors:** Tania Henriquez, Larissa Wirtz, Dan Su, Heinrich Jung

**Affiliations:** Microbiology, Department Biology 1, Ludwig Maximilians University Munich, D-82152 Martinsried, Germany; T.Henriquez@bio.lmu.de (T.H.); larissa.wirtz@biologie.uni-muenchen.de (L.W.); D.Su@biologie.uni-muenchen.de (D.S.)

**Keywords:** secondary transport, solute/sodium symport, SLC5, PutP, signal transduction, bacterial two-component systems, bacterial sensor kinase

## Abstract

The solute/sodium symporter family (SSS family; TC 2.A.21; SLC5) consists of integral membrane proteins that use an existing sodium gradient to drive the uphill transport of various solutes, such as sugars, amino acids, vitamins, or ions across the membrane. This large family has representatives in all three kingdoms of life. The human sodium/iodide symporter (NIS) and the sodium/glucose transporter (SGLT1) are involved in diseases such as iodide transport defect or glucose-galactose malabsorption. Moreover, the bacterial sodium/proline symporter PutP and the sodium/sialic acid symporter SiaT play important roles in bacteria–host interactions. This review focuses on the physiological significance and structural and functional features of prokaryotic members of the SSS family. Special emphasis will be given to the roles and properties of proteins containing an SSS family domain fused to domains typically found in bacterial sensor kinases.

## 1. Introduction

Inwardly directed electrochemical sodium ion gradients are important parts of bioenergetic circuits in pro- and eukaryotic cells. The idea that these gradients play a role in the active transport of solutes originally came from Robert K. Crane. He proposed at a Symposium on Membrane Transport and Metabolism in Prague in 1960 that the transport of sodium ions and glucose are coupled (Na^+^/glucose cotransport hypothesis) [1,2,3]. Peter Mitchel later generalized the idea of ion-coupled substrate transport and coined the term *symport* for processes in which coupling ion and substrate are transported in the same direction across the membrane [4].

In prokaryotes, the sodium ion gradients are established by primary sodium pumps (for example, sodium pumping complexes of the respiratory chain [5,6], membrane-integrated decarboxylases [7], sodium-translocating ATPases [8], and sodium/proton antiporters [9]. The electrochemical sodium ion gradients can be used by secondary transporters to drive the transport of solutes across membranes. These transporters are classified into families based on sequence similarities and common functional features. The glycoside-pentoside-hexuronide (GPH)/cation symporter family (TC2.A.2), the betaine-choline-carnitine-choline transporter (BCCT) family (TC2.A.15), the solute/sodium symporter (SSS) family (TC2.A.21), the neurotransmitter/sodium symporter (NSS) family (TC2.A.22), the dicarboxylate-amino acid-cation symporter (DAACS) family (TC2.A.23), and the glutamate/sodium symporter (ESS) family (TC2.A.27) are examples for families containing well-characterized sodium-dependent transporters of prokaryotic origin (http://www.tcdb.org, accessed on 30 November 2020) [10].

In recent years, crystallization-based structural analyses revealed important insights into the three-dimensional structures of various transporters including sodium-dependent systems. The analyses led to the discovery of common structural folds (for example, MFS fold, LeuT fold) [11,12]. Thereby, common core structures are shared even by transporters that do not have a significant sequence similarity or fulfill different functions (for example, sodium or proton-coupled solute uptake or solute antiport). In some cases, relatively small changes (for example, a positively charged amino acid in place of a site of sodium binding) can lead to a fundamental change in the coupling mechanism [13]. The structural insights led to a reclassification of the transporters. For example, the BCCT, SSS, and NSS families share, together with the amino acid-polyamine-organocation (APC) family and other transporter families, the LeuT fold and are now grouped together to constitute the APC superfamily [14,15]. Furthermore, transporters sharing a common core structure were eventually crystallized in different conformations. Comparison of these conformations provided insights into different conformational states underlying the respective transport cycle [16,17,18]. Analyses of conformational dynamics of transporters were further advanced by site-directed labeling combined with fluorescence or electron paramagnetic resonance (EPR) spectroscopic approaches [19,20,21,22,23]. More recently, single-molecule Förster resonance energy transfer (smFRET) and simulations of molecular dynamics were used to probe conformational dynamics of transporters under biologically relevant conditions [18,24,25]. These and other investigations of structure-function relationships in transporters greatly advanced our understanding of molecular details of solute transport across membranes.

Here, we briefly summarize information on the physiological significance, structure, and molecular mechanisms of function of transporters of the SSS family focusing on the prokaryotic part of the family. In addition to transport proteins, members of the SSS family show a distant similarity to the N-terminal domains of some sensor kinases of bacterial two component signal transduction pathways [26,27]. Initially it was not clear whether the SSS domain of the sensor kinases controls the kinase activity (for example, whether it is required for signal perception and transduction of the signal to other domains of the protein) and/or also functions as a solute transporter. In recent years, some of these sensor kinases and the functionally associated response regulators were characterized. Here, special emphasis will be given to the distribution, physiological significance, and functional properties of these prokaryotic two-component systems.

## 2. Functional Properties and Physiological Significance of Prokaryotic Transporters of the SSS Family

Transporters of the SSS family are responsible for the supply of cells with nutrients (for example, monosaccharides and amino acids), vitamins, and anions [26]. For eukaryotic SSS family members, the role in the development of diseases has been intensively investigated. For example, mutations in genes of human sodium/glucose transporters (SGLTs) cause the rare diseases glucose-galactose malabsorption and familial renal glucosuria [28]. Substrates of the sodium/multivitamin transporter (SMVT) play central roles in the cellular metabolism, and transporter defects can lead to growth retardation, dermatological disorders, and neurological disorders [29]. The human sodium/iodide symporter (NIS) is required for hormone synthesis in the thyroid and used for diagnostics and therapy of thyroid cancer [30]. Prokaryotic SSS family members catalyze the uptake of organic compounds as carbon, nitrogen and energy sources, and contribute to the adaptation of cells to environmental stresses such as osmotic stress and oxidative stress. Some of the SSS-related proteins perform important functions in regulating the metabolism of bacteria. Fulfilling these transport and regulatory functions, the SSS family members contribute also to the virulence of a variety of human pathogens. Selected examples are discussed in the following subsections.

### 2.1. PutP-Mediated Nutrient Supply and Osmoadaptation

**Figure 1:**
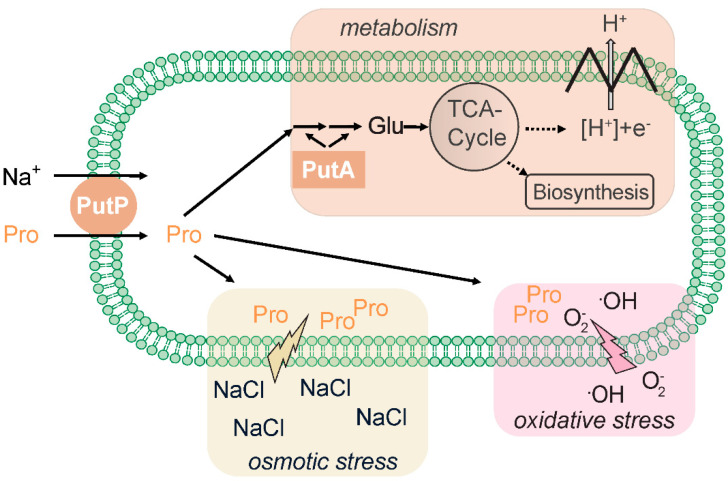
Possible roles of PutP in the proline metabolism of prokaryotes.

The SSS family transporter PutP catalyzes the sodium ion-dependent uptake of the amino acid proline in cells [31]. Similar to SGLT, both sodium ions and a membrane potential are crucial for substrate accumulation by PutP [2,32]. PutP has a high affinity for proline and is highly specific for the amino acid [32,33] (Table 1). The transporter is widespread in the prokaryotic world and can be found in archaea (for example, in the orders Methanococcales, Archaeoglobales, Thermococcales, Halobacteriales) as well as in Gram-positive and Gram-negative bacteria [26]. In *Enterobacteriaceae*, PutP is associated with PutA, a multifunctional proline dehydrogenase that oxidizes proline via ∆^1^-pyrroline-5-carboxylate and L-glutamate-γ-semialdehyde to glutamate [34]. The latter amino acid is central to the carbon and nitrogen metabolism and is further converted for catabolic and anabolic purposes (Figure 1). For example, amino transferases can transfer the amino group from glutamate to α-ketoacids, yielding other amino acids, and the citric acid cycle intermediate α-ketoglutarate, or glutamate, can be oxidized by glutamate dehydrogenase to yield α-ketoglutarate and ammonia. Other bacteria and archaea possess similar degradation pathways, although regulation and enzymatic basis of proline oxidation varies between organisms [35,36,37,38,39]. Proline accumulation via PutP can also play a role in the adaption of cells to osmotic stress. For example, transcription of the gene encoding the PutP ortholog OpuE in *Bacillus subtilus* is upregulated by means of sigma A- and sigma B-dependent stress-responsive promoters [40,41]. However, contrary to other proline and betaine transporters such as ProP (major facilitator superfamily [42]) and BetP (betaine/carnitine/choline transporter family [43], the activity of PutP and its orthologs is not stimulated at the protein level [44].

Furthermore, intracellular accumulation of l-proline can protect mammals, plants, fungi, yeast, and bacteria from damage by reactive oxygen species (for example, ·OH, ^1^O_2_) [45]. The protective effect may rely on the unique chemical properties of l-proline (for example, secondary amine of the pyrrolidine ring and low ionization potential) and involve the chemical modification of l-proline by reactive oxygen species [46,47]. On the other hand, up-regulation of *putA* may lead to the generation of reactive oxygen species, thereby decreasing oxidative resistance of bacteria and other organisms [45,48].

The amino acid proline plays an important role in interactions between pathogens and hosts [49]. In fact, disruption of PutP-dependent proline uptake attenuates the virulence of different pathogens [31]. For example, proline transport is vital for the survival and growth of *Staphylococcus aureus* upon human infection [50]. In this context, the *putP* gene is transcriptionally activated by low-proline and high osmotic environments in murine and human clinical specimens [51]. PutP is thought to particularly stimulate the early stages of the infection process by helping the pathogen to adapt to high osmolarity conditions in the host [50,51,52]. In addition, since many *S. aureus* strains are proline auxotrophs, PutP is required to supply the bacterium with the proteinogenic amino acid [51]. Despite the high similarity to *E. coli* PutP and the conservation of amino acids known to be involved in sodium binding in solute/sodium symporters, the activity of *S. aureus* PutP was not stimulated by NaCl [53,54]. Therefore, it has been suggested that under high osmolarity conditions, PutP-mediated proline uptake in *S. aureus* might be driven by a proton motive force instead of a sodium motive force [50]. More investigations at the biochemical level are necessary to test the coupling mechanism.

*Helicobacter pylori*, a causative agent of stomach inflammation and cancer [55], needs PutP for the successful colonization of the stomach of Mongolian gerbils [56]. Since the *putP* gene is associated with *putA* encoding proline dehydrogenase [49], the supply of the bacterium with proline as a carbon, nitrogen, and/or energy source seems to be of particular significance under the conditions in the stomach. This idea is supported by the observation that proline is the predominant amino acid in the gastric juice of humans infected with *H. pylori* [57]. Whether PutP contributes also to adaption to high osmolarity conditions (for example in the mucus layer of the stomach) requires further investigations. *H. pylori* PutP is sodium-dependent, and its functional properties are almost identical to the *E. coli* ortholog [58].

Furthermore, proline is known to be involved in regulating alternative lifestyles of *Photorhabdus luminescens*, a γ-proteobacterium that lives in symbiosis with nematodes and is a lethal pathogen of insects [59]. By accumulating proline in the cells, PutP contributes to an enhanced production of selected secondary metabolites known to be involved in antibiosis, insect virulence, and nematode mutualism [60]. Finally, PutP was shown to affect pulmonary and systematic infections of mice by the Gram-negative bacterium *Francisella novicida* [61], and facilitates the colonization of the plant rhizosphere by *Pseudomonas putida* [39].

### 2.2. Sugar Uptake via SSS Family Transporters

Glucose is an important carbon and energy source for pro- and eukaryotic cells. While in mammalian cells, glucose is coupled to sodium ions and catalyzed by SSS family transporters of the SGLT group [2], many bacteria take up glucose via a phosphotransferase system (PTS) which transports and modifies glucose to glucose-6-phosphate [65]. However, bacteria need to adapt to rapidly changing environments, and therefore often employ several transporters of different families (ABC, MFS, SSS, PTS) for the same substrate [65]. For example, the marine bacterium *Vibrio parahaemolyticus* can take up glucose (galactose) via a PTS and by vSGLT (SglS), a sodium-dependent SSS family protein [66] (Table 1). Proteins similar to SGLT are predicted based on genome analyses also for other bacteria including, for example, the marine bacterium *Rhodopirellula baltica,* the Gram-positive soil bacterium *Streptomyces coelicolor*, the gut bacterium *Bacteroides plebeius*, and the Gram-negative bacterium *Teredinibacter turnerae* that lives in symbiosis with mollusks (string-db.org). Similar to glucose (galactose), the monosaccharide mannose can be taken up by a phosphotransferase system (ManP^I^) and by an SSS transporter, the putative sodium/mannose transporter (ManP^II^) as suggested for the marine bacteria of the genus *Shewanella* [67]. Whether this and other related proteins indeed catalyze the sodium-coupled uptake of the respective monosaccharide still has to be tested biochemically. In any case, in the fight against bacterial multidrug resistance, vSGLT was recently discussed to represent a drug target [68].

SSS proteins also play an important role for the uptake of sialic acids, a family of 9-carbon sugar acids found predominantly on the cell-surface glycans of humans and other animals [69]. Mammalian commensals and pathogenic bacteria that colonize sialic acid rich tissues, such as the respiratory or gastrointestinal tract, have evolved mechanisms to use host-derived sialic acids [70]. Bacteria can employ different types of transporters for the uptake of the sugar acids. One of these transporters is the SSS protein SiaT (sometimes also termed NanT, which should not be confused with the MFS transporter NanT of *Escherichia coli* and its orthologs) (Table 1). Orthologs of SiaT are found, for example, in *Vibrio fischeri* and *Lactobacillus sakei*, and in the human pathogens *Salmonella enterica* serovar Typhimurium, *S. aureus,* and *Clostridium difficile* [69]. In the case of the latter two pathogens, SiaT (NanT) proved to be important for the colonization of the mouse intestine after treatment with antibiotics [71].

### 2.3. Uptake of Short Chain Organic Acids via SSS Family Transporters

Short organic acids are produced and secreted by pro- and eukaryotes and play important roles in bacteria–host interactions and interactions within bacterial communities, for example, in a process called cross-feeding [72,73,74]. SSS transporters such as ActP (sodium/acetate symporter) [75], MctC (proton/pyruvate, propionate, acetate symporter) [76] and MctP (cation/lactate, pyruvate, propionate, butyrate symporter) [77] contribute to the dynamics of these interactions by catalyzing the uptake of the respective short chain organic acid from the environment. ActP is found in many bacteria, including *Enterobacteriaceae* and *Pseudomonadaceae*. Interestingly, acetate uptake and metabolism in *Pseudomonadaceae* and other γ-Proteobacteria are controlled by a two-component signal transduction system with a senor kinase containing an SSS family domain (see below). In addition to acetate transport, ActP has been shown to catalyze the uptake of toxic tellurite [78].

## 3. Structural Basis of Transport

**Figure 2:**
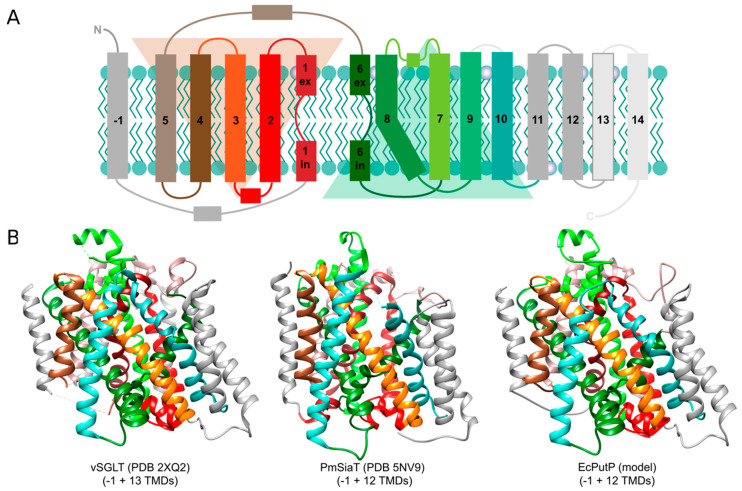
(**A**) Membrane topology of solute/sodium symporter (SSS) family transporters. SSS transporters are composed of 13 to 15 transmembrane domains (TMDs) connected by hydrophilic loops. The N terminus of the transporters is located on the outer site of the membrane. The transporters share the LeuT-type structural fold that is characterized by a core of ten TMDs that are arranged in five + five inverted repeats with an antiparallel orientation and a pseudosymmetry axis in the membrane plane [89]. The core domain starts at TMD 2 of SSS transporters (TMD 1 of SSS transporters is counted as TMD -1. (**B**) Ribbon representation of the three-dimensional structures of vSGLT, PmSiaT and EcPutP. The structures of vSGLT [84] and PmSiaT [64] are the result of X-ray analyses of respective crystals. The structure of EcPutP is based on homology modeling and experimental distance restraints obtained by site-directed spin labeling and EPR spectroscopy [90]. The figures were generated using UCSF Chimera. Chimera is developed by the Resource for Biocomputing, Visualization, and Informatics at the University of California, San Francisco [91]. (**A**) was taken and modified from Jung et al., 2012 [31].

SSS transporters are composed of about 500 to 700 amino acids that form 13 (bacterial PutP, ActP, SiaT, MctP; human NIS and SMVT), 14 (bacterial and human SGLT), or 15 (bacterial ManP^II^) transmembrane domains (TMDs) and hydrophilic loops connecting the TMDs on both sites of the membrane (www.uniprot.org, accessed on 1 February 2020). The *N* terminus of the transporters is located on the outer site of the membrane [79,80,81,82]. Crystal structures are available for SGLT of *V. parahaemolyticus* (vSGLT) [83,84] and SiaT of *Proteus mirabilis* (PmSiaT) [64]. The structural analyses revealed that SSS proteins share the same structural fold with the sodium-dependent leucine transporter LeuT of the thermophilic marine bacterium *Aquifex aeolicus* (AaLeuT, neurotransmitter/sodium symporter family, NSS, TC 2.A.22) [12,85]. The fold is characterized by a core of ten TMDs (cTMDs) that are arranged in five + five inverted repeats (in SSS transporters TMDs 2 to 6 and 7 to 12) with an antiparallel orientation and a pseudosymmetry axis in the membrane plane. To avoid confusion, TMD 1 of SSS transporters is counted as TMD -1 followed by the cTMDs 1 to 10 and more non-core TMDs in the *C*-terminal region of the transporters (Figure 2A). The TMDs are intertwined with cTMDs 1, 2, 3, 6, 7, 8 and 10 forming the central sites of substrate and sodium binding [12] (Figure 2B). Furthermore, cTMDs 1 and 6 contain unwound regions that are crucial for ligand binding and conformational alteration underlying the transport cycle. For the NSS transporter LeuT [86,87] and the SSS transporter PutP [20,88], features of the structural fold were confirmed by comprehensive electron paramagnetic resonance (EPR) spectroscopic analyses.

## 4. Molecular Mechanism of Transport

### 4.1. Sites of Substrate and Sodium Ion Binding

**Figure 3:**
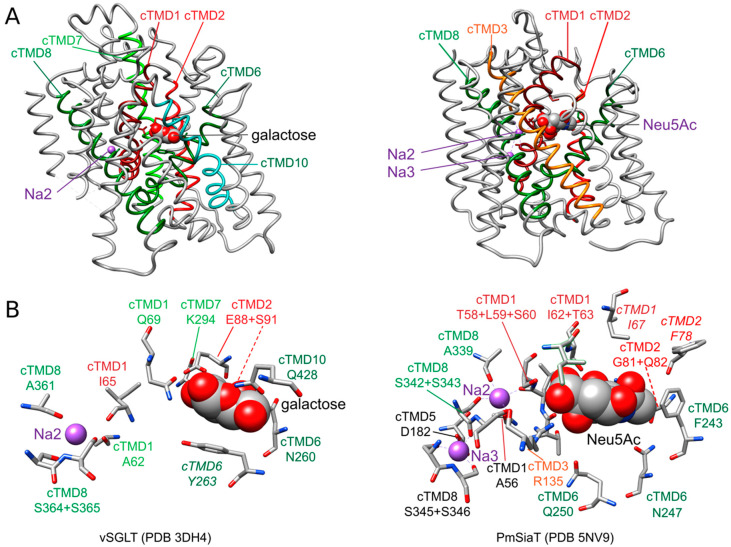
Sodium ion and substrate binding sites of vSGLT and PmSiaT. (**A**) Sideview of vSGLT and PmSiaT in the membrane plane. Core transmembrane domains (cTMDs) that coordinate with sodium ions and substrate are depicted in color, while the remaining TMDs are colored in grey. Substrates are shown as grey spheres colored by heteroatom and sodium ions are shown in purple. (**B**) Amino acids coordinating sodium ions or substrate. Amino acids are presented as grey sticks colored by heteroatom and substrates are presented as in (**A**). Amino acids positions highlighted in *italics* are considered to function as thin gates. Images were generated based on the crystal structures of vSGLT (3DH4) and PmSiaT (PDB 5NV9) using UCSF Chimera. Chimera is developed by the Resource for Biocomputing, Visualization, and Informatics at the University of California, San Francisco [91]. Neu5Ac, *N*-acetylneuraminic acid.

Sites of sodium ion binding in transporters were modeled based on the available crystal structures in combinations with amino acid substitution analyses. By this means, two putative sodium ion binding sites were identified in LeuT, Na1 and Na2 [85]. The central site of sodium ion binding (Na2) is highly conserved between transporters belonging to the structural class of proteins with a LeuT fold including SSS family transporters. It is located about half-way in the membrane between cTMDs 1 and 8 including the unwound region in cTMD1 (Figure 3A). Thereby, the sodium ion is coordinated by the main-chain carboxyl oxygen atoms of three nonpolar, aliphatic amino acids (two in cTMD1 and one in cTMD8) and the hydroxyl groups of two serine (or threonine) residues in cTMD8 [31,64,84] (Figure 3B and Table S1). In LeuT, a second sodium binding site (termed Na1 site) was suggested. The sodium ion at this site is proposed to participate directly in coordinating the substrate leucine [85].

A site corresponding to Na1 of LeuT has so far not been detected in SSS family members. However, the crystal structure of SiaT suggests a third site for sodium binding (termed Na3 site) that is located more towards the cytoplasmic side of the transporter, 0.65 nm away from the Na2 site. Here, the sodium ion is proposed to be coordinated by the carboxyl group of an acidic amino acid of cTMD5, a main-chain carbonyl oxygen and the hydroxyl groups of two serine of cTMD8 (Figure 3 and Table S1). While substitutions of amino acids of the putative Na2 site are highly deleterious for the function of all transporters investigated, substitutions at the proposed Na3 site are more nuanced. It has been suggested that sodium at this site plays a more modulatory role and helps to further pre-organize the substrate binding site by stabilizing the transporter in an outward-facing conformation [64]. The authors of the latter article suggested that SSS family transporters with a sodium: substrate stoichiometry of 2:1 (SiaT, human SGLT1) contain the Na2 and Na3 sites, while transporters with a 1:1 stoichiometry (PutP, vSGLT) harbor only the Na2 site. Nevertheless, an alignment of the amino acid sequences revealed that the Na3 site is in part conserved also in PutP (Figure S1). Substitution of respective amino acids in PutP (compare Table S1) affected transport properties similarly to as observed for SiaT [64,92,93,94]. Without a crystal structure at the time, the functional results obtained with PutP led to the conclusion that these amino acids are important for sodium release on the cytoplasmic side of PutP. In view of the new insights into the structure and function of SiaT, the role of the respective amino acids in PutP needs to be revisited.

Due to the chemical diversity of the substrates of SSS family proteins, substrate binding sites are much less conserved and more complex compared to sodium binding sites (Figure S1). Among different TMDs of the core structural motif participating in substrate binding, there are always amino acids of the unwound regions of cTMDs 1 and 6 that contribute to coordinating the substrate (Table S1). For example, binding of *N*-acetylneuraminic acid to SiaT is achieved by interactions with eight amino acids of cTMDs 1, 2, 3, and 6 and via seven water molecules. A hydration layer lies between the substrate and cTMDs 5 and 6 with hydrogen bonds to water and water-mediated interactions with side chains in cTMDs 2 and 6 [64] (Figure 3B).

There has been controversy regarding the existence of a second binding site (termed S2 site) in the NSS family protein LeuT located more externally above the central substrate binding site (termed S1) [95,96]. Substrate binding, flux analyses and computational studies suggest that the S2 site constitutes a high-affinity ligand binding site that is crucial for the transport cycle [95,97]. It has been hypothesized that the S2 site could allosterically modulate substrate release both positively and negatively. For example, binding of a second substrate molecule in S2 induces the release of the substrate bound to S1, whereas inhibitor binding prevents the release of substrate from S1 [98]. Based on the results with LeuT, the SSS family proteins vSGLT and PutP were examined for the existence of a second substrate binding site [99]. Radiolabeled galactose and proline saturation binding experiments indicated that both vSGLT and PutP can simultaneously bind two substrate molecules (Table S1). Amino acid substitutions in S1 or S2 reduced the binding capacity from two substrate molecules to one substrate molecule per transporter and impaired transport [99]. Furthermore, for vSGLT, the computational analyses suggest that the second binding site aligns to the S1 site of LeuT, while the amino acid coordinating galactose on the crystal structure [83] forms the more external binding site (S2) [99]. However, emerging evidence suggests that SGLT, like the lactose permease LacY (major facilitator family), may contain only one sugar binding site [21,100,101]. In any case, more computational and functional analyses are necessary to elucidate the existence and precise functional role of a possible second substrate binding site in SSS family transporters.

### 4.2. Transport by Alternating Access

**Figure 4:**
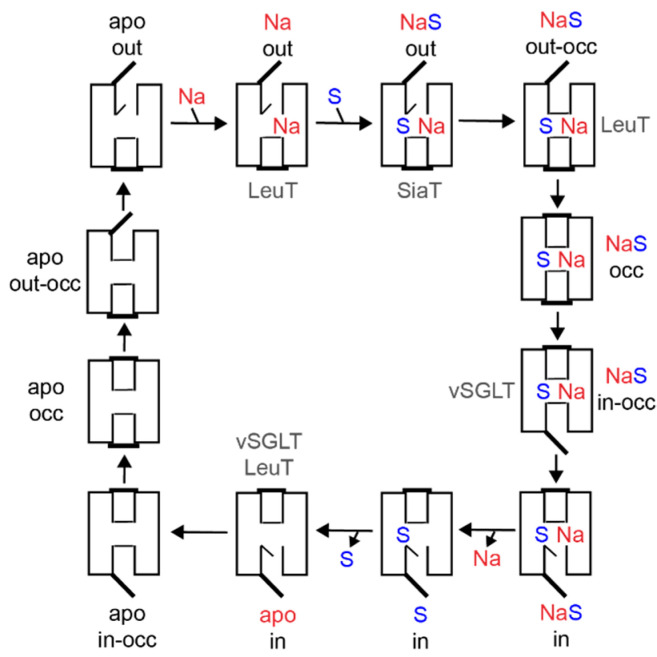
Model of conformational states underlying the alternating access mechanism of SSS family transporters and other transporters with a LeuT-type structural fold. The conformational states observed in crystal structures of vSGLT, PmSiaT and AaLeuT are indicated. Comprehensive kinetic analyses particularly with SGLT [2] and to some extent with PutP [106] propose an ordered binding mechanism. Sodium ions bind first to the transporter in the outward-open apo state inducing a conformational alteration that facilitates substrate binding. The ion-substrate-protein complex undergoes further conformational alterations that lead, via an occluded state, to an opening of the ion and substrate binding sites towards the inside of the cell, and finally, to the release of both ligands into the cytosol. The resulting inwardly oriented apo state of the transporters changes to the outward-open conformation to allow for a new transport cycle. Reciprocal opening and closing of inwardly and outwardly oriented cavities may involve movement of thin gates (vSGLT: Y263; PmSiaT: I67, F78; AaLeuT: Y108, F253), rearrangements of interactions between TMDs as well as between TMDs and inner and outer loops (for example the loop connecting TMDs 7 and 8 [11,64,84]. out: outward-open conformation, occ: occluded conformation, in: inward-open conformation.

Transporters have been proposed to function following an alternating access mechanism. Thereby, a centrally located substrate binding site is accessible either from the outside or from the inside [102,103,104]. The elucidation of the 3D structures of transporters of different (super)families in different conformational states over the last fifteen years has confirmed the correctness of the alternating access mechanism and revealed detailed mechanistic insights. The location of hydrophilic pathways (cavities) connecting the substrate binding site with either the outer environment or the cytosol, and of structural elements capable of blocking one pathway or the other (referred to as gates) were discovered. In addition to outward and inward open conformations, occluded states and intermediate states of gate opening and closure were identified (Figure 4). The alternating access mechanisms vary in detail depending on structural fold, substrate specificity, and mechanism of energization [105].

The SSS family transporter vSGLT has been crystallized in two conformations: (1) inward-occluded state with galactose bound to the center of the core domain (vSGLT-wild type, PDB: 3DH4) and (2) inward-open apo-state (vSGLT-K294A, PDB: 2XQ2) [83,84]. The pathway to the outside is blocked by a hydrophobic “thin” gate formed by M73 (cTMD1), Y87 (cTMD2) and F424 (outer end of cTMD10) and located directly above the central substrate binding site. In addition, interactions between the outer halves of cTMDs 1, 2, 6 and 10 and the loops connecting cTMDs 1 and 2, 7 and 8, and 9 and 10 (“thick” gate) prevent access to the central binding site. The hydrophilic pathway from the central substrate binding site to the cytoplasmic side of the transporter is (partially) open and lined by the inner portions of cTMDs 1, 2, 3, 6, and 8 [83,84]. In the inward-occluded state, access to the central substrate binding site is blocked by Y263 (cTMD6) that appears to function as an inner “thin” gate (Figure 3B). Comparison of the crystal structures, molecular dynamics simulation, and functional biochemical analyses led to the hypothesis that the transition from the outward- to the inward-occluded state of vSGLT alters the coordination of sodium at the Na2 leading finally to the release of sodium on the inner side of the transporter. Subsequent conformational changes including a movement of cTMD1 disrupt a hydrogen bond between N64 (cTMD1) and Y263 (cTMD6), allowing the side chain of Y263 to reorient and to open a pathway to the intracellular space. Additional rigid body movements then widen the inner pathway leading to substrate release into the cytosol [84].

PmSiaT has been crystallized in an outward-open conformation in complex with *N*-acetylneuraminic acid and two sodium ions (pdb: 5NV9) [64]. The open outer cavity is lined by cTMDs 1, 2, 3, 6, 8 and 10. The closed inner gate is stabilized by interactions between the inner portions of cTMDs 1, 6, 8 and 9 and the loops connecting cTMDs -1 and 1, and 4 and 5. Generation of an inward-open conformation based on vSGLT and morphing of both states suggest that the closing of the outer gate involves movements of the outer portion of cTMD10 towards cTMDs 1 and 2, cTMD9 towards cTMD2, and the outer loop connecting cTMDs 7 and 8 towards cTMD1. During the closing process, a “thin” gate is formed above the substrate binding site by the hydrophobic amino acids I67 (cTMD1), F78 (cTMD2) and W404 (outer end of cTMD10) [64] (Figure 3B). The amino acids align with the amino acids of the outer “thin” gate of vSGLT. To open the inner gate, interactions between the above listed inner portions of cTMDs and inner loops are disrupted. At the same time, cTMDs 8 and 9 move readily away from the inner pathway axis thereby providing access from the central binding site to the cytosol [64].

For PutP, comprehensive cysteine accessibility, fluorescence, and EPR spectroscopic analyses suggest that the transporter adopts an inward-open conformation in the absence of substrate (and a membrane potential) [80,88,94,107,108]. The inner portions of cTMDs 1, 6, and 8 have been suggested to line the inwardly-oriented hydrophilic pathway. Cysteine placed at various site in the inner cTMD portions were readily modified by sufhydryl reagents, and modification was blocked by the addition of a substrate in the presence of sodium. These results suggest that the inner portions of cTMDs 1, 6, and 8 participate in the inner gating mechanism [88,94,107]. Furthermore, D55 (cTMD1) and Y248 (cTMD6) proved crucial for PutP function. Since the amino acids align with N64 (cTMD1) and Y263 (cTMD6) of vSGLT, it was speculated that D55 and Y248 participate in the formation of an inner gate in PutP [94,109]. Out of the amino acids of PutP (L64, cTMD1; T83, cTMD2; and W405, outer end of cTMD10) that align with the three amino acids forming the outer “thin” gate in vSGLT [83], L64 and W405 proved to be crucial for proline transport and may participate in forming the outer gate in PutP (T83 was not investigated) [90,107]. Furthermore, the complete spin-labeling site scan of the extracellular loop connecting cTMDs 7 and 8 revealed that the loop participates in the closure of the outer pathway also in PutP [20]. The results suggest that F314 of the loop anchors the loop by means of hydrophobic contacts to cTMD1 close to the ligand binding sites. In addition, E311 at the tip of the loop, and various amino acids around the outer end of TM10’, proved particularly important for PutP functions, thereby interactions of E311 with the peptide backbone of cTMD10 might also stabilize the closed state [90].

The comparison of the gating mechanisms underlying alternating access in the different transporters reveals conserved feature and diverse differences. In all proteins investigated, cTMDs 1 and 6, and the flexibility of the unwound regions of the cTMDs, play a decisive role. In addition, the external loop connecting cTMDs 7 and 8 plays an important role in closing the outwardly-directed pathway. Interactions of the loop with cTMDs 1 and 10 stabilize the transporters in a conformational state that is closed to the outside. Finally, the participation of a hydrophobic “thin” gate above the central substrate binding side seems to be a common feature of SSS family transporters and other proteins with a LeuT structural motif.

## 5. SSS Domain-Dependent Sensor Kinases

### 5.1. Occurrence, Significance and Targets of SSS Domain-Dependent Signal Transduction Systems

Sensor kinases containing an SSS domain were first described in 2001 [110]. This domain is distantly related, but homologous to the PutP transporter of *E. coli* [26,111]. The predicted role of these proteins would be to act as sensors of membrane-associated stimuli such as ligand binding, turgor or mechanical stress of the membrane, and ion or electrochemical gradients and transport processes, among others [112]. However, to this day, the specific stimuli and roles of the SSS domain in sensor kinases are enigmatic. In this section, we provide an overview on the information available about the occurrence and distribution of the SSS domain in connection to other protein domains (such as in sensor kinases), and summarize the current knowledge on the biological role and target genes of the associated two-component system.

#### 5.1.1. Distribution in Prokaryotes

**Figure 5:**
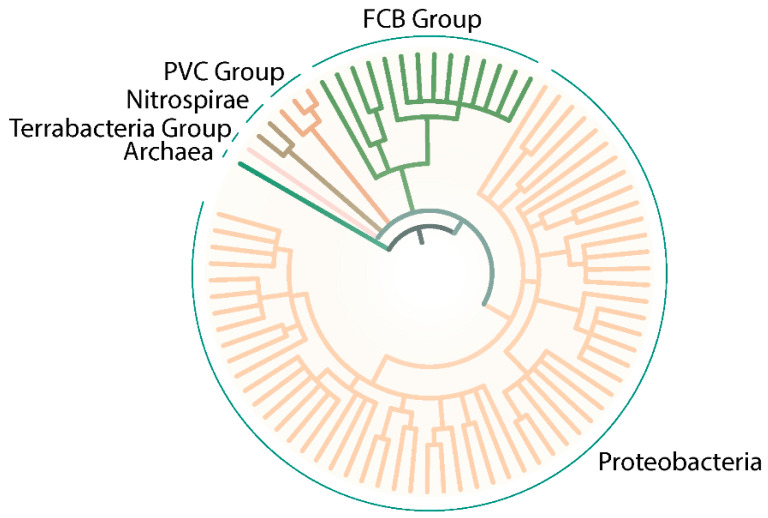
Prediction of organisms containing SSS family in association wih HisKa domain. SMART was used to identify proteins containing Pfam:ssf (SSS) and HisKa domains. After selection of representative organisms to be displayed for each group, a Newick formatted tree based on NCBI taxonomy was generated. The tree was visualized and modified using iTOL (Intreactive Tree Of Life) [116,117] tool and Adobe Illustrator.

Since the initial description of SSS domain-dependent sensor kinases, only a few dozen publications have appeared on this topic. In this context, most of the research has been published in Proteobacteria, specifically γ-Proteobacteria (such as *Vibrio* spp. or *Pseudomonas* spp.). However, the analysis of the SMART database (a Simple Modular Architecture Research Tool) [113,114,115] revealed a wide distribution through the Bacteria domain, including Proteobacteria, FCB group (Bacteroidetes), PVC group (Verrucomicrobia), and Nitrospirae and Terrabacteria groups (Deinococcus) (Figure 5). Surprisingly, even some Archaea are predicted to contain SSS domains in putative kinases (Figure 5).

**Figure 6:**
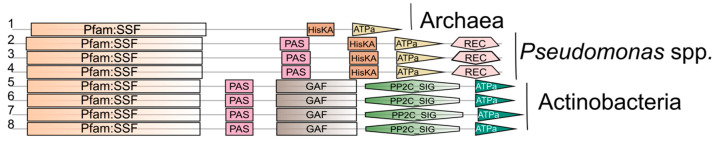
Gram-positive organisms containing SSS family domain associated to HATPase_c. SMART database was used to identify proteins containing Pfam:ssf and HATPase_c (presented in the figure as “ATPa”) domains. Four isolates of Actinobacteria (5: *Actinospica acidiphila*, 6: *Streptomyces griseorubens*, 7: *Streptomyces albus*, 8: *Streptomyces gilvosporeus*) were selected and, as a comparison, three *Pseudomonas* spp. (2: *Pseudomonas stutzeri*, 3: *Pseudomonas fluorescens* Q2-87, 4: *Pseudomonas corrugata*) and one Archaea were also analyzed (1: *Candidatus* Nitrosocaldus cavascurensis). The ID of the isolates (SMART database) can be found in Table S2.

At a first glance, the analysis indicated the absence of proteins containing Pfam:ssf (SSS) and HisKa domains in Gram-positive bacteria. Since the crystal structure of several sensor kinases revealed the importance of two different domains for their function: the ATPase domain, also called HATPase in Pfam (that binds ATP and transfer the γ-phosphate to the second domain), and the dimerization/phosphorylation domain, also called HisKA in Pfam (involved in phosphotransfer from the kinase to the response regulator) [118], a second analysis to identify proteins including SSS and HATPase_c (Histidine kinase-like ATPases) domains was performed. The results indicate the presence of proteins containing these domains in Actinobacteria (Figure 6). In this case, the proteins contained additional domains such as GAF and PP2C_SIG (Sigma factor PP2C-like phosphatases), which are also involved in signaling. These findings suggest the SSS family domain as a component of proteins involved in signal transduction in different groups of organisms, which would not always be linked to histidine kinase domain.

#### 5.1.2. Biological Significance and Targets of Two-Component Systems Containing SSS Domains

Microorganisms can live in extremely different and fluctuating environments, since they have developed numerous features to sense and respond to these conditions [119]. These mechanisms include the one-/two-/multi-component systems, which are named according to the number of proteins involved in the transduction process. In the following, information on the physiological role of recently characterized two-component systems with an SSS domain containing sensor kinase and a response regulator is summarized.

The CbrA/CbrB two-component system was originally described in 2001 in a variant of *Pseudomonas aeruginosa* PAO1 that could not utilize selected amino acids (such as histidine, arginine and proline), polyamines and agmatine as sole carbon or nitrogen source [110]. The gene encoding the sensor kinase of the system was designated as *cbrA*, for catabolite regulation, due to the pleiotropic effects produced by its interruption [110]. Further analyses revealed an involvement of the CbrA/CbrB system in carbon catabolite repression (CCR) [120,121]. Homologs of the system in *Pseudomonas fluorescens* [122] and *P. putida* [123,124] have similar functions in histidine metabolism and CCR. Additionally, the involvement of the system in virulence and antibiotic resistance was reported in *P. aeruginosa* [125,126]. CbrA/CbrB was also recently described in *Azotobacter vinelandii*, where it would be involved in glucose uptake (through the regulation of the GluP transporter) and alginate production [127,128].

Another well-described system in γ-Proteobacteria is CrbS/CrbR, also called MxtR/ErdR in *P. aeruginosa*. It has been linked to acetate metabolism based on analyses of the regulation of the *acs* gene and the growth behavior on acetate in *V. cholerae* [129,130,131], *P. aeruginosa* [130,132], *P. fluorescens* [133] and *P. entomophila* [130]. Several elements were reported to be regulated by this system, which range from other genes possibly involved in acetate metabolism, such as *actP* (encoding an acetate permease) or Pflu0110 (putative hydrolase) in *P. fluorescens* [133], to genes related to antibiotic resistance [132]. The involvement of this system in pathogenicity was studied in *V. cholerae*, where the expression of *crbS* or *acs*-1 was linked to the consumption of host intestinal acetate by the bacteria, which deactivated the insulin signaling and lipid accumulation in enterocytes. This mechanism led to the death of the host, *Drosophila melanogaster* [129]. In *Pseudomonas* spp., a role in virulence was suggested by Zaoui and colleagues in 2011 [132]. In their study, the authors described the expression of important elements for bacterial virulence such as quorum sensing molecules, pyoverdine and pyocyanin, among others, were affected by the presence of the sensor kinase MxtR [132]. However, to date, there are no in vivo results to quantify the importance of the system in pathogenicity.

In addition to the findings in other γ-Proteobacteria associated to CbrA/CbrB and MxtR/ErdR, Rodríguez-Moya and coworkers described in 2010 the presence of a two-component system in the obligately halophilic *Chromohalobacter salexigens*, which would be involved in osmoadaptation [134]. This system is composed of a response regulator (EupR) and a multi-sensor hybrid histidine kinase (the product of the gene *csal869* that includes a PutP/Pfam SSF domain). In the study, the authors reported the involvement of the system in the regulation of the utilization of ectoines as a carbon source and compatible solutes in this halophilic bacterium [135]. However, it is still unknown whether this system has a more general function regulating also other processes (in a similar way to CbrA/CbrB and MxtR/ErdR in virulence).

Additionally, a recent report described a two-component system, PrlS/PrlR, in *Brucella melitensis* (α-Proteobacteria) that would be important for bacterial adaptation to ionic strength and persistence in mice [136]. The sensor kinase of the system, PrlS, is a hybrid histidine kinase with an SSS N-terminal domain, while the response regulator, PrlR, belongs to the LuxR family. According to the authors, the signal sensed by this two-component system would be ionic strength [136]. In this context, it is interesting to notice that PrlS proteins from other organisms, such as *Aeromonas hydrophila*, were previously predicted to contain an SSS domain [26], which would suggest a role in *A. hydrophila* similar to the one in *B. melitensis*.

### 5.2. Structure-Functions Relationsships in SSS Domain-Dependent Sensor Kinases

#### 5.2.1. General Characteristics of SSS-Containing Sensor Kinases

**Figure 7:**
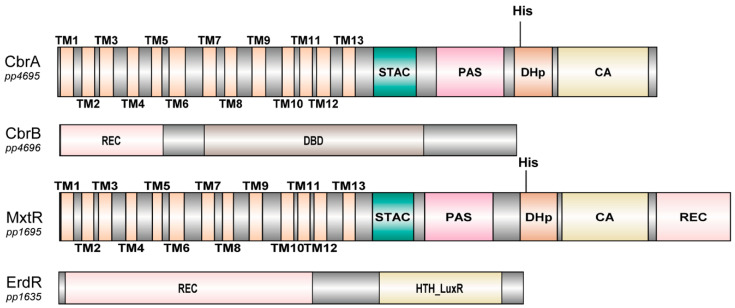
Domain structure of CbrA and MxtR as typical SSS-dependent sensor kinases with the according response regulators. The N-terminal portion of the sensor kinases forms 13 TMDs that resemble the topology of an SSS family member, while the C-terminal portions contains domains typically found in sensor kinases. TM, also called TMD, transmembrane domain; STAC, SLC and TCST-Associated Component; PAS, Per-Arnt-Sim; DHp, histidine phosphotransfer domain, also referred to as HisKA; CA, catalytic ATP-binding domain, also referred to as HATpase_c; REC, receiver; DBD, DNA binding domain (for “sigma-54 interaction domain” predicted by ScanProsite; HTH, helix-turn-helix. Images created with DOG 2.0 illustrator [140]. Domain prediction relies on SMART [113,114] and [133].

From the biochemical point of view, there are only two well-studied SSS-containing sensor kinases in γ-Proteobacteria: CbrA and MxtR (also known as CrbS), that were analyzed in *P. putida*, *P. fluorescens*, *P. aeruginosa*, *V. cholerae*, and *A. vinelandii* [110,123,124,127,128,130,133]. In both CbrA and MxtR, the N-terminal domain is predicted to form 13 TMDs resembling the topology of a member of the SSS family (Figure 7). Transport activity has only been shown so far for the SSS domain of CbrA, with the substrate being L-histidine [124,137]. Connected to this transporter protein is a STAC domain, that was only recently described as a new protein domain. The STAC domain could serve as a linker between the transporter and the C-terminal domains, that are typically found in histidine sensor kinases [133,138]. Following the STAC domain, a Per-Arnt-Sim (PAS) domain, a histidine phosphotransfer (DHp) domain, and a catalytic ATP-binding (CA) domain are present [124,133]. The only difference in domain structure between CbrA and MxtR is that MxtR carries at its C terminus a REC (receiver) domain that CbrA lacks, which implies that MxtR is a hybrid kinase that functions via a phosphorelay mechanism [139].

#### 5.2.2. Transport Activity of SSS Family-Containing Sensor Kinase CbrA

The transport activity of CbrA was analyzed in *P. fluorescens* [137] and *P. putida* [124]. CbrA was identified as a possible histidine uptake system in *P. fluorescens* SBW25, because a mutant lacking known histidine transporters was still able to survive with histidine as the sole C and N source, indicating the presence of another transporter. A transposon screen selecting for growth on histidine led to the identification of *cbrA*. Uptake of ^3^H-histidine by wild type cells and mutants confirmed that CbrA functions as a constitutive histidine transporter [137]. The role of the SSS domain in histidine uptake was further explored in a *P. putida* KT2440 mutant [124]. CbrA takes up L-histidine with a *k_m_* of 0.7 µM, similar to PutP of *E. coli* (Table 1). The maximum uptake rate is relatively low (0.27 nmol min^−1^ mg^−1^ of total cell protein). Interestingly, even though CbrA belongs to the sodium solute symporter family [10,26], not a sodium but a proton electrochemical gradient appears to stimulate transport. Amino acids that are involved in sodium binding (Na2 site) in PutP are not conserved in CbrA [124]. It was shown also that a mutant lacking the cytosolic domains of CbrA (SSS domain only) or a mutant that has no kinase activity (CbrA-H766N) transport histidine with the same efficiency, suggesting that the sensor kinase domain is not required for the transport function [124]. The specificity for histidine is high, and neither other amino acids nor histidine analogues affected the uptake of ^3^H-L-histidine in competition experiments [124].

#### 5.2.3. Properties of Domains Associated with SSS Domain-Containing Sensor Kinases

In prokaryotes, mostly dimeric histidine kinases autophosphorylate at a conserved histidine residue using ATP as a phosphate donor [141]. The H box, with the histidine residue that is the phosphorylation target in the DHp domain, as well as the N-, D- and G- boxes in the CA domain are highly conserved between different SSS domain-containing sensor kinases from different species. It was shown that CbrA contained in *E. coli* TKR2000 membranes can autophosphorylate upon the addition of radioactively labeled γ-^32^P-ATP [124]. The histidine residue that is phosphorylated is H766 at the beginning of the DHp domain. The CbrA variant H766N did not show phosphorylation. The protein could also be purified without the SSS domain (CbrA∆SSS). The remaining soluble sensor kinase was still phosphorylated at H766. In addition, a reporter assay showed that soluble CbrA∆SSS can activate expression of the downstream target gene *crcZ* [124]. In addition, in *P. fluorescens*, a chimeric construct consisting of the SSS domain of CrbS and the kinase domain of CbrA could be used to complement a *∆cbrA ∆crbS* mutant and support growth on histidine [133]. The results were confirmed in *P. putida* [124]. In conclusion, it seems that the SSS domain is not necessary for the kinase function of CbrA. Even though physical interaction between the two protein domains seems not to be essential for transport and signal transduction, the SSS domain modulates the auto kinase kinetics. This is corroborated by the fact that a target gene can be activated by a soluble CbrA variant (CbrA∆SSS) missing the TMDs, but only when it is expressed on a high level [123]. It was also shown that the phosphate can be transferred from CbrA to Asp52 in the REC domain of purified CbrB [124]. Phosphatase activity of CbrA, i.e., dephosphorylation of CbrB-P_i_, has not been observed so far.

The STAC domain was recently described either as a separate protein or embedded within proteins that combine SSS domains with sensor kinase domains [138]. The vast majority of STAC domains occurs between N-terminal SSS domains and C-terminal sensor kinase domains, but so far, the role of the STAC domain remains elusive. A CbrA∆STAC variant had no visible phenotype in *P. fluorescens* [133], but the expression of target gene *crcZ* was decreased in *P. putida* by the deletion of the STAC domain [124].

The structure of PAS domains is broadly conserved and comprises a five-stranded antiparallel ß-sheet and several α-helices, even though the sequence identity is below 20% on average [142]. Almost half of the described PAS containing proteins are histidine kinases, but effector domains include serine/threonine kinases, guanylate cyclases, phosphodiesterases, methyl-accepting chemotaxis proteins and more, that play a role in signal transduction [143]. Typically, the PAS domain is linked N-terminally to the effector domain, like in the case of CbrA or MxtR (CrbS), but there are also examples where the PAS domain is located C-terminally, e.g., in the Sim protein, which contains the first described PAS domain [144]. The PAS domain evolved to bind a huge variety of ligands and cofactors, which can serve as a direct signal or be a step-in sensing signals like gases, redox potential or light [143]. The range of ligands that can bind PAS domains is broad and includes hemes, flavins, amino acids, divalent metal cations, coumaric acid, and fatty acids. Described SSS domain-dependent kinases carry a conserved PAS domain between the STAC and DHp domain in the cytosolic part of the protein. The fact that a CbrA∆SSS variant can still function as a kinase and induce signal transduction makes it likely that the sensed signal is intracellular [123,124]. The PAS domain is a likely candidate for a yet to be identified signal. The idea is confirmed by the observation that a CbrA∆PAS variant was not able to induce expression of one of its target genes [123].

MxtR (CrbS) of *P. aeruginosa* was cloned as a truncated version without TMDs and used in auto phosphorylation assays. The phosphorylation was inhibited in vitro by ubiquinone, the central electron carrier of respiration, indicating a possible role of MxtR (CrbS) in sensing the redox state of the cell [132]. As mentioned before, the only obvious difference between these sensor kinases is related to the REC domain. A REC (receiver) domain is usually found in response regulators, which is the case for CbrB and ErdR, next to one or several effector domains [112]. Its role is to receive the phosphate from the histidine kinase on an aspartate residue and through a neighboring effector domain leading to a cellular response, usually a change in transcription of target genes. If a REC domain occurs in a hybrid sensor kinase, like MxtR (CrbS), this is called a phosphorelay mechanism. These systems provide greater versatility in signaling strategies and occur often in eukaryotes, while prokaryotes mostly use simpler schemes [139]. So far, the role of MxtR’s REC domain is unknown.

## 6. Conclusions

The crystal structures of SGLT of *V. parahaemolyticus* and SiaT of *P. mirabilis* show that the SSS family transporters also share the structural fold of the NSS transporter LeuT, despite the lack of similarity at the amino acid sequence level. Clearly, 3D structures of non-sugar transporters such as the amino acid transporter PutP or vitamin transporters would complete the picture. Numerous structure-function analyses have provided important insights into the details of the transport cycle of SSS family proteins, including the location of sites of sodium and substrate binding, and conformational changes associated with the alternating access mechanism of transport. Future research should focus on more detailed information on physiological relevant intermediate states of transporters, the kinetics of the transitions between these states, the precise stoichiometry of coupling ion and substrate translocated per transporter molecule, and the role of proposed secondary ligand binding sites. This aim can be achieved by combining structure determination, spectroscopic approaches including single molecule analyses, kinetic measurements, and computational simulations. In fact, recent computational analyses of vSGLT suggested, for example, a previously unknown proofreading/editing mechanism enabling the bacteria to discriminate between glucose and potentially toxic analogs [145]. To better understand the role of SSS domains in sensor kinases of regulatory two-component systems, high resolution structures and more precise information on the functions of individual domains (for example, identification of ligands and of the functional consequences of ligand binding and potentially transport) and on interactions between these domains are required. In addition, more effort is necessary to explore the physiological relevance of these systems in microorganisms. Finally, the knowledge on the structure, functions, and dynamics of the transporters and transporter-related signal transduction systems will provide tools to modulate transporter activities for therapeutic purposes and biotechnological applications.

## Figures and Tables

**Table 1:** Functional properties of PutP, vSGLT, and SiaT.

Property	PutP [32,62]	vSGLT [63]	SiaT [64]
Substrate and apparent *k*_M_	l-proline 2 µM	Galactose 158 µM	*N*-acetylneuraminic acid 16 µM
Apparent *k*_sodium_	31 µM	129 mM	n.d.
Sodium: substrate stoichiometry	1:1	1:1	2:1

## Data Availability

Data sharing not applicable.

## Supplementary Materials

Supplementary materials can be found at https://www.mdpi.com/1422-0067/22/4/1880/s1. Figure S1: Amino acid sequence alignment of the SSS family transporters SGLT of *V. parahaemolyticus* (vSGLT), SiaT of *P. mirabilis* (PmSiaT) and PutP of *E. coli* (EcPutP), Table S1: Amino acids involved in sodium and substrate binding in PutP, vSGLT and SiaT, Table S2: Information of the isolates from the SMART database used for Figure 5.
