# Supplementary material for: Prokaryotic Solute/Sodium Symporters: Versatile Functions and Mechanisms of a Transporter Family †
Source: Int J Mol Sci. 2021 Feb 13;22(4):1880. doi: 10.3390/ijms22041880 (PMC7918813; doi:10.3390/ijms22041880)
Supplement: Supplementary file 1 [file ijms-22-01880-s001.pdf]

Supplementary Information

Prokaryotic solute/sodium symporters: versatile functions and mechanisms of a transporter family

Tania Henriquez [1], Larissa Wirtz [1], Dan Su [1] and Heinrich Jung [1,*]

[1] Microbiology, Dept. Biology 1, Ludwig Maximilians University Munich, D-82152 Martinsried, Germany
* Correspondence: hjung@lmu.de; Tel.: +49-89-218074630

```
                         ncTMD-1                            cTMD1a
VcSglT    MSNIEHGLSFIDIMVFAIYVAIIIGVGLWVSRDKKGTQKSTEDYFLAGKSLPWWAVGASL      60
PmSiaT    --MQLHDFGFINYAVLFGYLAAMLLVGVYFSK----RQKTADDYFRGGGRVPGWAAGVSV      54
EcPUTP    -----MAISTPMLVTFCVYIFGMILIGFIAWR----STKNFDDYILGGRSLGPFVTALSA      51
                 :.     .:  *:  :: :*.    :      *. :**: .*  :  :... *

                    cTMD1b                  cTMD2
VcSglT    IAANISAEQFIGMSGSGYSIGLAI-------ASYEWMSAITLIIVGKYFLPIFIEKGIYT      113
PmSiaT    FATTLSSITFMSIPAKAYTSDWTF-------IIGQYLAIAILPLVFYFYIPFFRKLKITS      107
EcPUTP    GASDMSGWLLMGLPGAVFLSGISESWIAIGLTLGAWINWKL--VAGRLRVHTEYNNNALT      109
           *: :*.  ::.: .  :  . :          ::      :.    :    :    :

                                     cTMD3                   cTMD4
VcSglT    IPEFVEKRFNKKLKTILAVFWISLYIF--VNLTSVLYLGGLALETILGIPLMYSILGLAL      171
PmSiaT    AYEYLEARFDVRSRLFASLS-FMLFHI--GRVAIITYLTVLALRPFMGIDPVVLIVLISL      164
EcPUTP    LPDYFTGRFEDKSRILRIISALVILLFFTIYCASGIVAGARLFESTFGMSYETALWAGAA      169
            ::.   **: : : :   :  : :   :     :          :.   :*:      :   :

                                  cTMD5
VcSglT    FALVYSIYGGLSAVVWTDVIQVFFLVLGGFMTTYMAV-SFIGGTDGWFAGVSKMVDAAPG      230
PmSiaT    LCIIYTWMGGIEGVIWTDVIQGLLLSGGAVLIFIMICFKVDGGISEIFTTTAQADK----      220
EcPUTP    ATILYTFIGGFLAVSWTDTVQASLMIFALILTPVIVIISV-GGFGDSLEVIKQKSI---E      225
            ::*:   **: .* ***.:*   ::   . .:   :    .. **  .   :     :

                                cTMD6a           cTMD6b
VcSglT    HFEMILDQSNPQYMNLPGIAVLIGGLWVANLYYWGFNQYIIQRTLAAKSVSEAQKGIVFA      290
PmSiaT    FFPT---TQWRWSWTDSTIPVLMIGFLFANIQQFTASQDVVQRYIVTDSIKETKRTLITN      277
EcPUTP    NVDML------KGLNFVAI----ISLMGWGLGYFGQ-PHILARFMAADSHHS----IVHA      270
           .            .   *      .:    .:  :      :: *  :.:.*   .     ::

              cTMD7
VcSglT    AFLKLIVPFL----VVLPGIAAYVITSDPQLMASLGDIAATNLPSAANADKAY-PWLTQF      345
PmSiaT    AKLVAIIPIF----FFAIGSALFVYYQQNP----------SLLPAGFNTGGILPLFIVTE      323
EcPUTP    RRISMTWMILCLAGAVAVGFFGIAYFNDHPALAGAV---------NQNAERVFIELAQIL      321
            :     ::      .   *    .   .:                       *:

                            cTMD8
VcSglT    LPVGVKGVVFAALAAAIVSSLASMLNSTATIFTMDIYKEYISPDSGDHKLVNVGRTAAVV      405
PmSiaT    MPIGIAGLIIAAIFAAAQSSISSSLNSISSCFNSDIYTRLSKSSPSPEQKMKVAKLVIIV      383
EcPUTP    FNPWIAGILLSAILAAVMSTLSCQLLVCSSAITEDLYKAFLRKHASQKELVWVGRVMVLV      381
          :   : *::::*: **  *:::. *   :: :. *:*.        . .: : *.:  :*

            cTMD9                           cTMD10
VcSglT    ALIIACLIA--P---MLGGIGQAFQYIQEYTGLVSPGILAVFLLGLFWKKTTSKGAIIGV      460
PmSiaT    AGIFSSLAAIWL---VLSDEAEIWDAFNSLIGLMGGPMTGLFMLGIFVKRANAGSAVVGI      440
EcPUTP    VALVAIALAANPENRVLGLVSYAWAGFG-------AAFGPVVLFSVMWSRMTRNGALAGM      434
          . :.:    *       :*.  .  :  :           :  :.::.:: .: .   .*: *:

              ncTMD11                     ncTMD12
VcSglT    VASIPFALFLK-FMPLS-MPFMDQMLYTLLFTMVVIAFTSL------------STSINDD      506
PmSiaT    IVSIIAVLAAR-YGSDL-----NFFFYGVIGSMSVVIAGTI------------TAPLF-A      481
EcPUTP    IIGALTVIVWKQFGWLGLYEIIPGFIFGSIGIVVFSLLGKAPSAAMQKRFAEADAHYHSA      494
          : .   .:  : :           :::  :  : .     .                :

VcSglT    DPKGISVTSSMFVTDRSFNIAAYGIMIVLAVLYTLFW      543
PmSiaT    PAKQLSLDDSET----SEN------------------      496
EcPUTP    PPSRLQES-----------------------------      502
            . :.
```

Supplementary Figure S1. Amino acid sequence alignment of the SSS family transporters SGLT of *V. parahaemolyticus* (vSGLT), SiaT of *P. mirabilis* (PmSiaT) and PutP of *E. coli* (EcPutP). The alignment was performed with Clustal Omega [1]. Amino acid sequences forming TMDs are underlined. TMDs are numbered as described in Figure 2a. Amino acids highlighted in red coordinate sodium at the Na2 site, while amino acids in orange belong to the recently described Na3 site of PmSiaT. Amino acids labeled in blue constitute the central substrate binding site. Amino acids highlighted in blue and *italic* interact with the substrate via water molecules. Amino acids highlighted in brown form the outer "thin" hydrophobic gate. For PutP, amino acids of particular functional significance are highlighted in **bold** (compare also Table 2). Information was taken from the following references: vSGLT [2,3], PmSiaT [4], EcPutP [5-9].

Suplementary Table S1. Amino acids involved in sodium and substrate binding in PutP, vSGLT and SiaT[a]

Function	PutP	vSGLT	SiaT
Na2 site	cTMD1: A53, M56<br>cTMD8: A337, S340, T341	cTMD1: A62, I65<br>cTMD8: A361, S364, S365	cTMD1: A56, L59<br>cTMD8: A339, S342, S343
Na3 site	cTMD5: D187 (?)<br>cTMD8: S340 (?), C344 (?), Q345 (?)	no experimental evidence	cTMD5: D182<br>cTMD8: S342, S345 S346
central substrate binding site	cTMD1: S54, S57 (?)<br>cTMD6: Y248, P252 (?)<br>cTMD8: C344, M369 (?)	cTMD1: Q69<br>cTMD2: E88, S91<br>cTMD6: N260<br>cTMD7: K294<br>cTMD10: Q428	cTMD1: T58, S60, T63<br>cTMD2: F78, Q82<br>cTMD3: R135<br>cTMD6: N247, Q250
2nd substrate binding site[b]	cTMD1: S57, W59 (?)<br>cTMD6: W244, Y248 (?)<br>cTMD10: L398, S402 (?)	cTMD1: S66 (?)<br>cTMD6: Y269, R273 (?)<br>cTMD8: S365, S368 (?)	no experimental evidence

[a]The data of the sodium and the central substrate binding sites of vSGLT and PmSiaT were taken from the analyses of respective crystal structures [4,10]. For PutP, the amino acids proposed to be involved in sodium and substrate binding were identified by amino acid replacements in combination with comprehensive analyses of transport kinetics, ligand affinities and site directed labeling approaches [5-8]. The location of a 2nd substrate binding site in PutP and vSGLT was predicted based on computational analyses in combination with amino acid replacements, substrate binding and transport studies [11].

[b]Of note: Structural alignments between LeuT and vSGLT revealed that the crystallographically identified galactose-binding site in vSGLT [10] is located in a more extracellular location relative to the central substrate-binding site in LeuT. Therefore, the existence of an additional galactose-binding site in vSGLT was suggested and experimentally tested that aligns to the central binding site of LeuT [11]. Following this logic, the amino acids of vSGLT listed in the table under "2nd substrate binding site" constitute the central binding site while the amino acids listed under "central binding site" form a more external binding site.

Supplementary Table S2. Information of the isolates from the SMART database used for Figure 5.

**Number in Figure 5**	**Isolates (Organisms/SMART ID)**
**1**	***Candidatus* Nitrosocaldus cavascurensis/ A0A2K5ARA0_9ARCH (A0A2K5ARA0)**
**2**	***Pseudomonas stutzeri*/ A0A0H3Z2P6_PSEST (A0A0H3Z2P6)**
**3**	***Pseudomonas fluorescens Q2-87*/J2EGW4_PSEFL (J2EGW4)**
**4**	***Pseudomonas corrugata*/ A0A1B3C9J6_9PSED (A0A1B3C9J6)**
**5**	***Actinospica acidiphila*/ UPI00052494D7**
**6**	***Streptomyces griseorubens*/ UPI00056BF26D**
**7**	***Streptomyces albus*/ UPI000689E04F**
**8**	***Streptomyces gilvosporeus*/ A0A1V0TJU0_9ACTN (A0A1V0TJU0)**

References

1. Madeira, F.; Park, Y.m.; Lee, J.; Buso, N.; Gur, T.; Madhusoodanan, N.; Basutkar, P.; Tivey, A.R.N.; Potter, S.C.; Finn, R.D., et al. The EMBL-EBI search and sequence analysis tools APIs in 2019. *Nucleic Acids Research* **2019**, *47*, W636-W641, doi:10.1093/nar/gkz268.
2. Watanabe, A.; Choe, S.; Chaptal, V.; Rosenberg, J.M.; Wright, E.M.; Grabe, M.; Abramson, J. The mechanism of sodium and substrate release from the binding pocket of vSGLT. *Nature* **2010**, *468*, 988-991, doi:10.1038/nature09580.
3. Faham, S.; Watanabe, A.; Besserer, G.M.; Cascio, D.; Specht, A.; Hirayama, B.A.; Wright, E.M.; Abramson, J. The crystal structure of a sodium galactose transporter reveals mechanistic insights into Na^+/sugar symport. *Science* **2008**, *321*, 810-814, doi:10.1126/science.1160406.
4. Wahlgren, W.Y.; Dunevall, E.; North, R.A.; Paz, A.; Scalise, M.; Bisignano, P.; Bengtsson-Palme, J.; Goyal, P.; Claesson, E.; Caing-Carlsson, R., et al. Substrate-bound outward-open structure of a Na^+-coupled sialic acid symporter reveals a new Na^+ site. *Nat Commun* **2018**, *9*, 1753, doi:10.1038/s41467-018-04045-7.
5. Hilger, D.; Böhm, M.; Hackmann, A.; Jung, H. Role of Ser-340 and Thr-341 in transmembrane domain IX of the Na+/proline transporter PutP of *Escherichia coli* in ligand binding and transport. *Journal of Biological Chemistry* **2008**, *283*, 4921-4929, doi:10.1074/jbc.M706741200.
6. Quick, M.; Jung, H. A conserved aspartate residue, Asp187, is important for Na^+-dependent proline binding and transport by the Na^+/proline transporter of *Escherichia coli. Biochemistry* **1998**, *37*, 13800-13806, doi:10.1021/bi980562j.
7. Pirch, T.; Quick, M.; Nietschke, M.; Langkamp, M.; Jung, H. Sites important for Na^+ and substrate binding in the Na^+/proline transporter of *Escherichia coli*, a member of the Na^+/solute symporter family. *J Biol Chem* **2002**, *277*, 8790-8796, doi:10.1074/jbc.M111008200.
8. Olkhova, E.; Raba, M.; Bracher, S.; Hilger, D.; Jung, H. Homology model of the Na^+/proline transporter PutP of *Escherichia coli* and its functional implications. *J Mol Biol* **2011**, *406*, 59-74, doi:10.1016/j.jmb.2010.11.045.

9. Bracher, S.; Schmidt, C.C.; Dittmer, S.I.; Jung, H. Core transmembrane domain 6 plays a pivotal role in the transport cycle of the sodium/proline symporter PutP. *The Journal of biological chemistry* **2016**, *291*, 26208-26215, doi:10.1074/jbc.M116.753103.
10. Faham, S.; Watanabe, A.; Besserer, G.M.; Cascio, D.; Specht, A.; Hirayama, B.A.; Wright, E.M.; Abramson, J. The crystal structure of a sodium galactose transporter reveals mechanistic insights into Na^+/sugar symport. *Science* **2008**, *321*, 810-814.
11. Li, Z.; Lee, A.S.; Bracher, S.; Jung, H.; Paz, A.; Kumar, J.P.; Abramson, J.; Quick, M.; Shi, L. Identification of a second substrate-binding site in solute-sodium symporters. *J Biol Chem* **2015**, *290*, 127-141, doi:10.1074/jbc.M114.584383.
